# iTRAQ-based quantitative proteomics analysis of rice leaves infected by *Rice stripe virus* reveals several proteins involved in symptom formation

**DOI:** 10.1186/s12985-015-0328-y

**Published:** 2015-06-26

**Authors:** Biao Wang, Jamal-U-Ddin Hajano, Yingdang Ren, Chuantao Lu, Xifeng Wang

**Affiliations:** State Key Laboratory for Biology of Plant Diseases and Insect Pests, Institute of Plant Protection, Chinese Academy of Agricultural Sciences, 100193, Beijing, China; Institute of Plant Protection, Henan Academy of Agricultural Sciences, 450002 Zhengzhou, China

**Keywords:** Rice, Proteome, iTRAQ, Magnesium chelatase, Peptidase, Plant defense

## Abstract

**Background:**

Rice plants infected by *Rice stripe virus* (RSV) usually leads to chlorosis and death of newly emerged leaves. However, the mechanism of RSV-induced these symptoms was not clear.

**Methods:**

We used an iTRAQ approach for a quantitative proteomics comparison of non-infected and infected rice leaves. RT-qPCR and Northern blot analyses were performed for assessing the transcription of candidate genes.

**Results:**

As a whole, 681 (65.8 % downregulated, 34.2 % upregulated infected vs. non-infected) differentially accumulated proteins were identified. A bioinformatics analysis indicated that ten of these regulated proteins are involved in chlorophyll biosynthesis and three in cell death processes. Subsequent RT-qPCR results showed that downregulation of magnesium chelatase was due to reduced expression levels of the genes encoding subunits CHLI and CHLD, which resulted in chlorophyll reduction involved in leaf chlorosis. Three aspartic proteases expressed higher in RSV-infected leaves than those in the control leaves, which were also implicated in RSV-induced cell death. Northern blot analyses of CHLI and p0026h03.19 confirmed the RT-qPCR results.

**Conclusions:**

The magnesium chelatase and aspartic proteases may be associated with RSV-induced leaf chlorosis and cell death, respectively. The findings may yield new insights into mechanisms underlying rice stripe disease symptom formation.

**Electronic supplementary material:**

The online version of this article (doi:10.1186/s12985-015-0328-y) contains supplementary material, which is available to authorized users.

## Background

*Rice stripe virus* (RSV), a member of the genus *Tenuivirus*, is one of the most economically important viruses in eastern Asia including China, Korea, and Japan [1]. In 1964, RSV was reported for the first time in Zhejiang Province [2] and then spread to 18 provinces in rice-growing areas of China [3]. From 2000 to 2005, 1,700,000 ha of rice fields were affected by this virus in Jiangsu Province, including 1,000,000 ha area where incidence was so severe that yield losses exceeded 50 %, and in some places no rice was harvested [4].

RSV is transmitted predominantly in a persistent propagative manner by the small brown planthopper (SBPH; *Laodelphax striatellus* Fallen) [5] and can be transmitted transovarially for more than 40 generations [6]. RSV has four single-stranded RNA segments, named RNA 1, 2, 3 and 4 in order of their molecular weight. Among these, RNA 3 encodes a nucleocapsid protein (NCP) from the viral complementary RNA [7], while RNA 4 encodes a disease specific protein (SP) from the viral RNA [8]. RSV-induced symptoms of rice typically are chlorotic stripes and mottlings on the leaves. Newly emerged leaves exhibit yellow stripes or necrosis, then folding and twisting; plants are stunted and finally dead [1].

Leaf chlorosis in general is widely accepted as a sign of reduction in chlorophyll [9, 10], and leaf chlorosis upon virus infection is also related to decreased chlorophyll [11]. Subsequent studies have shown that various molecular mechanisms are involved in leaf chlorosis during virus infection. For example, during *Cucumber mosaic virus* (CMV) infection, the expression of the genes encoding magnesium chelatase is regulated by CMV satellite RNA, thus blocking chlorophyll biosynthesis [12, 13]. In addition, chlorotic symptoms induced by *African cassava mosaic virus* (ACMV) are linked to the expression level of chlorophyll-related genes encoding proteins such as chlorophyllide a and chlorophyllide b [14]. However, the chlorosis on tobacco leaves during the flavum strain of *Tobacco mosaic virus* (TMV) infection not resulted from the reduction of chlorophyll biosynthesis, but was reduction of the core complexes of photosystem II and the oxygen evolving complex [15]. In a recent report, RSV SP interacted with PsbP (an oxygen-evolving complex protein) resulting in the downregulation of PsbP in chloroplasts, and then modulating RSV symptoms through disruption of chloroplast structure and function [16]. Whether other chlorophyll relation proteins are modulated during RSV infection has not been known.

In addition, if the cultivar is susceptible to RSV infection, newly emerged rice leaves usually exhibit necrosis [1]. Previous report indicated that a vacuolar processing enzyme that has caspase protease activity was indispensable for the TMV-induced hypersensitive response, which involves programmed cell death in tobacco [17]. Even in an uninfected healthy plant, the expression of aspartic proteases induces programmed cell death, and then involves in senescence [18]. Nevertheless, we still need to elucidate how the expression of aspartic proteases is regulated after RSV infection. Therefore, the key rice protein(s) involved in RSV-induced disease symptom formation require(s) further exploration.

Some techniques have been shown as powerful tools for understanding plant-pathogen interactions, including yeast two-hybrid system [19–21], glutathione-S transferase pull-down assay [22, 23], immunofluorescence laser scanning confocal microscopy [24, 25], 2D gel-based technology [26, 27], and iTRAQ (isobaric tag for relative and absolute quantitation) LC-MS/MS (liquid chromatography tandem mass spectrometry) technology [28]. iTRAQ LC-MS/MS technology adopted stable isotope labeling strategies of proteins or peptides for measurement and allowed relative quantitation comparison using an internal reference, and could simultaneously label and accurately quantify proteins from multiple samples [29, 30]. In this study, by using an iTRAQ-based quantitative proteomics approach, we analyzed protein accumulation profiles of RSV-infected leaves in comparison with healthy leaves to explore symptom formation and to understand rice-RSV interactions.

## Results

### Symptom formation and RT-PCR confirmation of infection

**Fig. 1:**
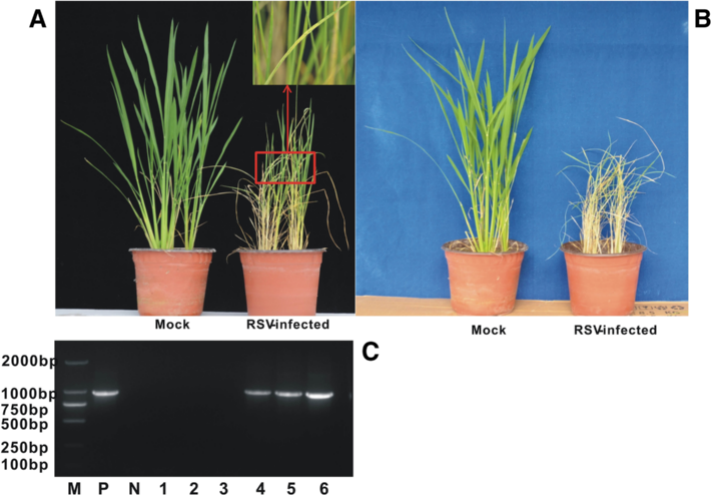
Typical chlorotic stripes and necrosis symptoms in rice plants induced by *Rice stripe virus* (RSV) and infection confirmation by RT-PCR detection. Both RSV-infected and mock rice plants were shown at (**a**) 21 days post inoculation (dpi) and (**b**) 23 dpi; (**c**) RT-PCR confirming that 969-bp fragment for RSV nucleocapsid gene (*NCP*) was absent in mock and present in symptomatic RSV-infected plants. Mock rice had typical healthy growth, but RSV-infected leaves showed typical chlorosis at 21 dpi (corner of panel **a**), and the entire plant was dead at 23 dpi (**b**). M, DL2000 DNA marker; P, RSV infected sample as positive control; N, healthy sample as negative control; 1-3, mock leaves; 4-6, RSV-infected leaves

There were 10 viruliferous SBPH allowed to feed on each plant of cv. Aichiasahi for 2-day inoculation access period. Newly emerged leaves on the initially inoculated plant developed pale-yellow stripes, which then collapsed in the form of blotches at 21 days post inoculation (dpi) (Fig. 1a). At 23 dpi, severe necrosis resulted in plant death (Fig. 1b). No disease symptoms were observed on mock plants. Samples of RSV-infected plants and control plants that were collected at 21 dpi to confirm infection by RT-PCR yielded an expected 969-bp fragment that was also found in a previously confirmed-positive sample (Fig. 1c). The 969-bp fragment was not present in the mock control or no-template control (NTC).

### Protein identification and quantification

**Fig. 2:**
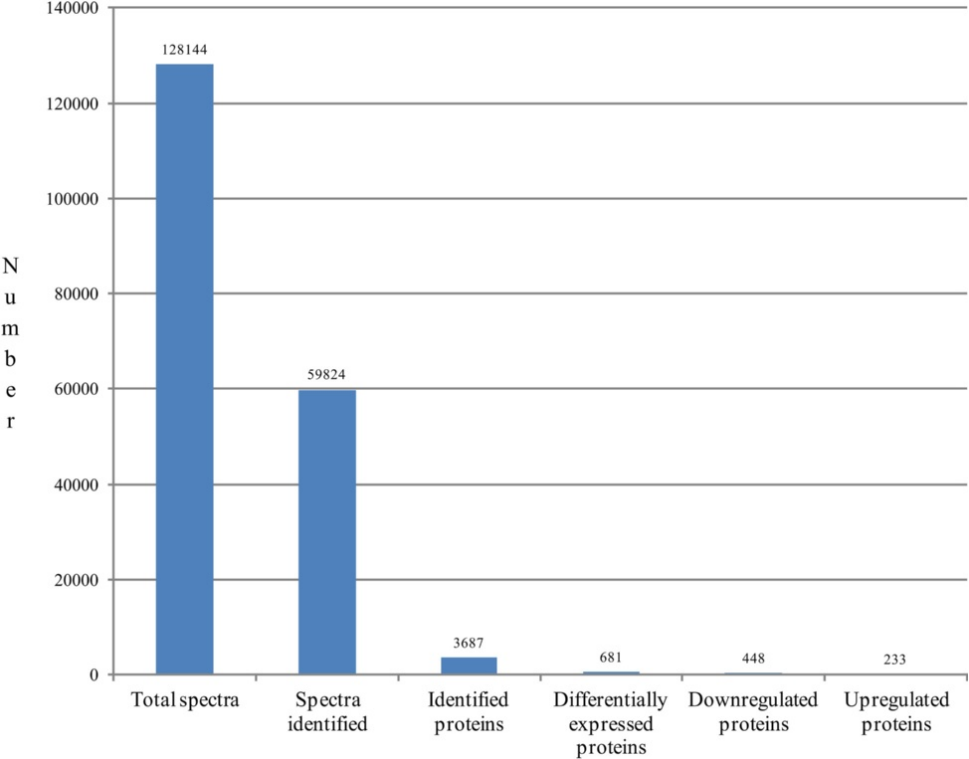
Statistics for total spectra for reversed-phase HPLC and LC-MS/MS, identified proteins and differentially accumulated proteins from iTRAQ proteomics by searching and analysis of NCBI database. Spectra scan ranged from 350 to 1800 m / z. Number refer to statistics of different parts: total spectra were generated from the iTRAQ experiment using the materials (RSV-infected and mock leaves); spectra identified matched known spectra; proteins identified analyses were determined by spectra identified upon the NCBI database; differentially accumulated proteins analysis based on the fold-change >1.5 or <0.667 (*P* < 0.05); downregulation proteins with fold-change >1.5 (*P* < 0.05); upregulation proteins with fold-change <0.667 (*P* < 0.05)

When the iTRAQ approach was used to analyze proteins obtained from RSV-infected leaves and mock leaves which were collected at 21 dpi, 128,144 spectra were totally obtained from an ABI-5600 system and then approximately 59,824 MS spectra identified matched known spectra. Overall, 3687 different proteins were identified when a false discovery rate (FDR) <1 % was applied to the dataset (Fig. 2). A total of 681 proteins were differentially accumulated, with a fold-change >1.5 (*P* < 0.05); 448 were downregulated, and 223 had a fold-change <0.67 (*P* < 0.05) (Table 1).

**Table 1:** Summary of the proteins identified by iTRAQ as being differentially accumulated in RSV-inoculated plants compared with mock-inoculated rice plants at 21dpi

Regulation	No. of proteins	David	GO^a^	Categories^b^	Percentage^c^	No. of functional groups
Down	448 (65.8 %)	332	317	203 BP	61.1	53
				154 CC	46.4	20
				233 MF	70.2	33
				129 KEGG	38.9	16
		unknown 116				
Up	233 (34.2 %)	178	175	112-BP	62.9	17
				64 CC	36.0	13
				125 MF	70.2	16
				7 -KEGG	39.9	13
		unknown 55				
Total	681					

Note: Using the David platform, 332 downregulated and 178 upregulated proteins were analyzed, and 317 and 175 proteins were annotated by GO, respectively. Annotated proteins were clustered by groups based on the BP, CC, MF and KEGG analyses

^a^GO annotation: BP, biological process; CC, cellular component; MF, molecular function

^b^Categories based on BP, CC, MF and KEGG

^c^Percentage of total proteins annotated

### Bioinformatics analysis

**Fig. 3:**
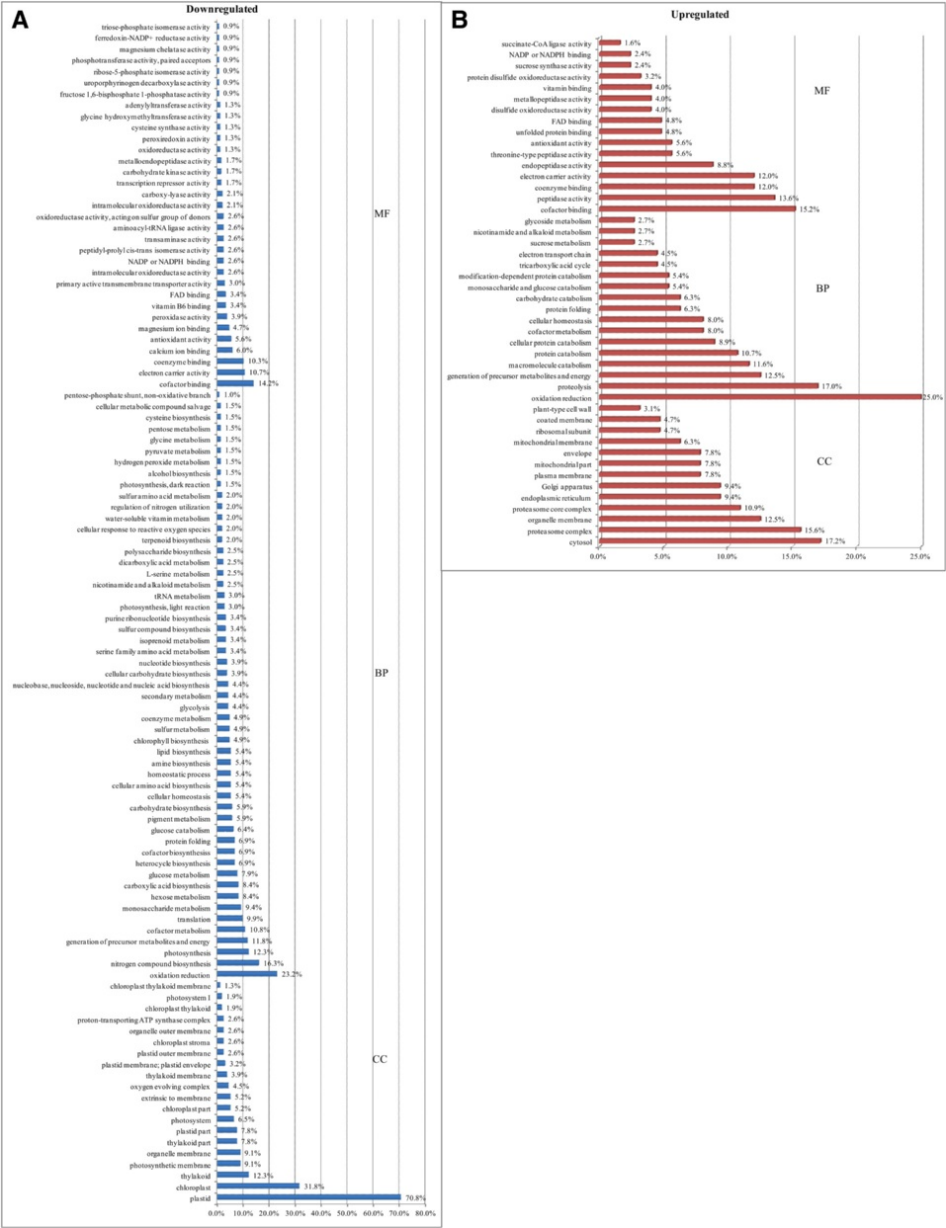
Gene Ontology enrichment analysis of differentially accumulated proteins from RSV-infected leaves compared with mock leaves. **a** Downregulated differentially accumulated proteins were annotated among 33 groups for molecular function (MF), 53 for biological process (BP) and 20 for cellular components (CC), respectively; **b**, Functional grouping of upregulated differentially accumulated proteins: 16 for MF, 17 for BP and 13 for CC

The identified and quantified proteins were then analyzed for function, pathway and interaction network. In the GO analysis, 358 proteins were involved in molecular function, 233 (70.2 %, 35 functional groups) were downregulated and 125 (70.2 %, 16 functional groups) were upregulated (Table 1, Additional file 1: Table S1). The molecular function of downregulated proteins was mainly in cofactor binding (14.2 %), electron carrier activity (10.7 %), coenzyme binding (10.3 %), calcium ion binding (6.0 %), antioxidant activity (5.6 %), magnesium ion binding (4.7 %), peroxidase activity (3.9 %), vitamin B6 binding (3.4 %), FAD (flavin adenine dinucleotide) binding (3.4 %), and primary active transmembrane transporter activity (3.0 %) (Fig. 3a, Additional file 1: Table S1). Upregulated proteins were involved in cofactor binding (15.2 %), peptidase activity (13.6 %), coenzyme binding (12.0 %), electron carrier activity (12.0 %), endopeptidase activity (8.8 %), threonine-type peptidase activity (5.6 %), antioxidant activity (5.6 %), unfolded protein binding (4.8 %), FAD binding (4.8 %), and disulfide oxidoreductase activity (4.0 %) (Fig. 3b, Additional file 1: Table S1). Peptidase activity, the largest group within the catalytic activity group, comprised metallopeptidase activity, aspartic-type endopeptidase, cysteine-type peptidase activity, serine-type peptidase activity. Biological process was influenced by 315 proteins, 203 (61.1 %, 53 functional groups) downregulated proteins which mostly were involved in oxidation reduction (23.2 %), nitrogen compound biosynthesis (16.3 %), photosynthesis (12.3 %), generation of precursor metabolites and energy (11.8 %), cofactor metabolism (10.8 %), translation (9.9 %), monosaccharide metabolism (9.4 %), hexose metabolism (8.4 %), carboxylic acid biosynthesis (8.4 %), glucose metabolism (7.9 %) (Fig. 3a, Additional file 1: Table S1). The other 112 (62.9 %, 17 groups) upregulated proteins were mostly involved in oxidation reduction (25.0 %), proteolysis (17.0 %), generation of precursor metabolites and energy (12.5 %), macromolecule catabolism (11.6 %), protein catabolism (10.7 %), cellular protein catabolism (8.9 %), cofactor metabolism (8.0 %), cellular homeostasis (8.0 %), protein folding (6.3 %), and carbohydrate catabolism (6.3 %) (Fig. 3b, Additional file 1: Table S1). Cellular components that were downregulated included 154 proteins (46.4 %, 20 component groups), located in the plastid (70.8 %), chloroplast (31.8 %), thylakoid (12.3 %), photosynthetic membrane (9.1 %), organellar membrane (9.1 %), thylakoid part (7.8 %), plastid part (7.8 %), photosystem (6.5 %), chloroplast part (5.2 %), extrinsic to membrane (5.2 %), and oxygen evolving complex (4.5 %) (Fig. 3a, Additional file 1: Table S1). The 64 (36.0 %, 13 component groups) upregulated proteins were located in the cytosol (17.2 %), proteasome complex (15.6 %), organelle membrane (12.5 %), proteasome core complex (10.9 %), endoplasmic reticulum (9.4 %), Golgi apparatus (9.4 %), envelope (7.8 %), mitochondrial membrane (6.3 %), ribosomal subunit (4.7 %), membrane coat (4.7 %), and cell junction (3.1 %) (Fig. 3b, Additional file 1: Table S1).

**Fig. 4:**
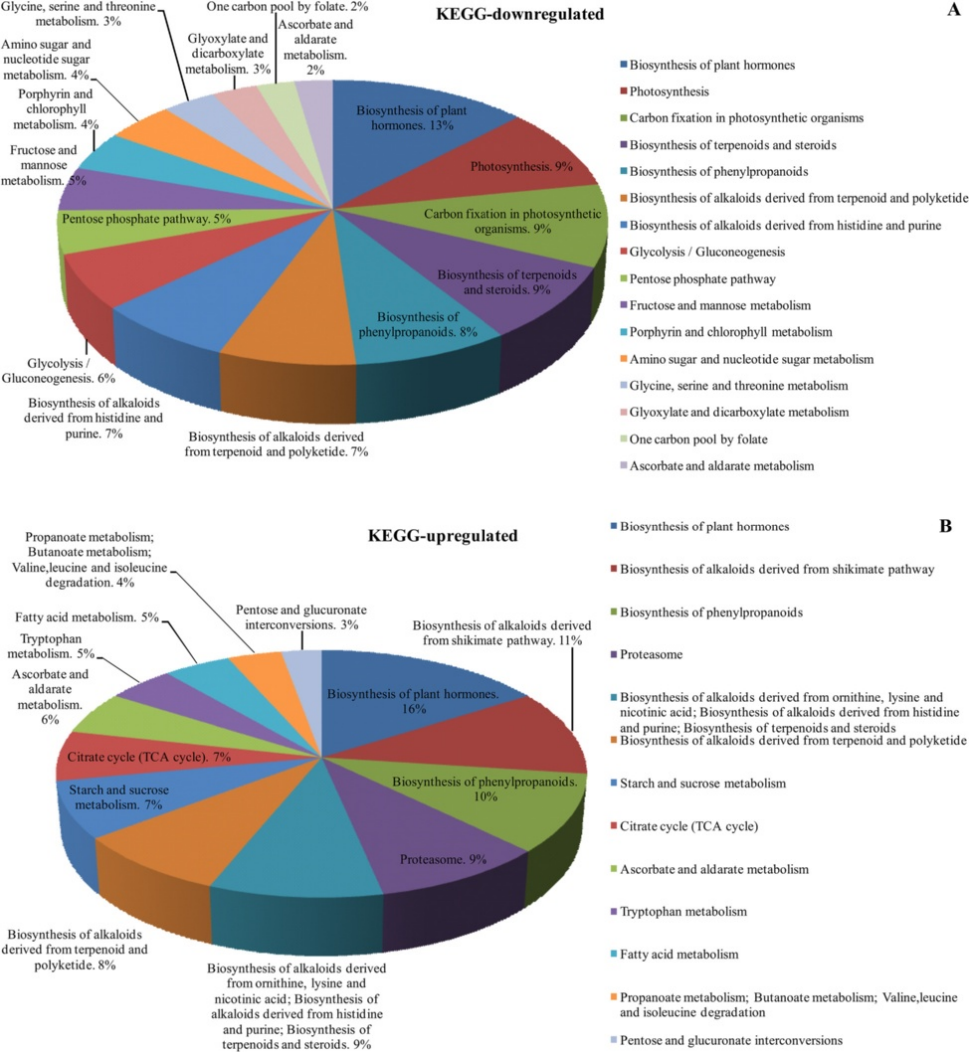
Kyoto Encyclopedia of Genes and Genomes (KEGG) pathways of differentially accumulated proteins that are identified from mock leaves and RSV-infected leaves for (**a**) downregulated and (**b**) upregulated. **a** downregulated proteins were annotated and participated in 16 pathways; (**b**) upregulated proteins were classified 13 pathways

The KEGG pathway analyses indicated that among the downregulated proteins, 13 % were involved in the biosynthesis of plant hormones; 9 % in photosynthesis, carbon fixation in photosynthetic organisms, biosynthesis of terpenoids and steroid; and 4 % in porphyrin and chlorophyll metabolism (Fig. 4a). However, among the upregulated proteins, 16 % were involved in biosynthesis of plant hormones, 11 % in biosynthesis of alkaloids derived from shikimate pathway, 10 % in biosynthesis of phenylpropanoids, and 9 % in proteasome, starch and sucrose metabolism, citrate cycle, tryptophan metabolism, fatty acid metabolism, propanoate metabolism, and pentose and glucuronate interconversions (Fig. 4b). When the identified proteins were analyzed with the STRING software, the results showed that 547 proteins were interacting with each other. In the constructed interaction network (Additional file 2: Figure S1), the proteins were roughly divided into three groups: metabolism (B), chloroplast (C) and defense (D).

### Proteins differentially accumulated in response to RSV infection

#### Metabolism group

Functions of the down- and up-regulated differentially accumulated metabolism group of proteins included monosaccharide metabolism, disaccharide metabolism, polysaccharide metabolism, generation of precursor metabolites and energy, amino acid metabolism, fatty acid metabolism, phosphorus metabolism, and sulfur metabolism. Basically, carbohydrate metabolism provided more suitable source of energy and carbon for plant development. For example, glyceraldehyde-3-phosphate dehydrogenase (GAPDH; 115458768, 115450493) and fructose-bisphosphate aldolase (115484401, 115468886, 115434198) were two important metabolic enzymes in glycolysis and gluconeogenesis [31]. Notable, evidences increasingly support the nonglycolytic functions of GAPDH, including apoptosis, DNA and RNA replication, DNA repair, RNA exportation, RNA synthesis, immunity response to various pathogens [32–38]. GAPDH strong binding of negative strand *Tomato bushy stunt virus* (TBSV) was key regulatory step to promote asymmetric RNA synthesis, so GAPDH played a role in viral RNA replication and RNA synthesis [34]. However, GAPDH preferentially binds positive strand *Bamboo mosaic virus* (BaMV), and it negatively regulated the accumulation of BaMV [35]. Additionally, GAPDH negatively regulate autophagy interaction with host protein and immunity-associated cell death and defense on TMV infection [38]. GAPDH may be involved in viral replication and defense during RSV infection. Proteins that decreased in expression belonged to the vitamin, nucleotide, isoprenoid, phosphorus, sulfur and cofactor metabolism groups, suggesting that RSV infection inhibited their expression (Table 2). Thus, numerous biological processes helped rice to counteract RSV invasion.

**Table 2:** Differentially accumulated proteins between mock-inoculated leaves and RSV-infected leaves

Accession number	Protein name categorized by process	Cov (95)	Number of Matching Peptides	Ratio	*P*-Value
	Chlorophyll biosynthetic process				
115453785	Magnesium-chelatase subunit ChlI, chloroplastic	46.7	29	17.5	4.50 × 10^-8^
115438661	Uroporphyrinogen decarboxylase 1, chloroplastic	14.9	8	13.4	2.85 × 10^-2^
115444475	Porphobilinogen deaminase, chloroplastic	51.1	21	11.7	4.50 × 10^-4^
115456135	Magnesium-chelatase subunit ChlD, chloroplastic	27.8	23	9.4	5.19 × 10^-7^
115477483	Glutamate-1-semialdehyde 2,1-aminomutase, chloroplastic	34.7	26	5.3	2.85 × 10^-2^
115452897	Uroporphyrinogen decarboxylase 2, chloroplastic	36.4	21	5.0	1.83 × 10^-2^
115436038	Protoporphyrinogen oxidase, chloroplastic	21.3	12	4.8	3.07 × 10^-4^
115435974	Magnesium-protoporphyrin IX monomethyl ester [oxidative] cyclase	42.2	25	3.7	1.16 × 10^-5^
115469822	Delta-aminolevulinic acid dehydratase, chloroplastic	29.1	16	3.5	4.11 × 10^-4^
115482796	Glutamyl-tRNA reductase, chloroplastic	16.4	9	3.4	1.34 × 10^-3^
	Photosynthesis				
109156602	Ribulose bisphosphate carboxylase large chain	82.2	508	44.1	1.71 × 10^-5^
115472625	Oxygen-evolving enhancer protein 3	41.5	57	31.9	3.46 × 10^-5^
115436780	Putative 33 kDa oxygen evolving protein of photosystem II	59.2	119	28.3	2.30 × 10^-10^
115470529	Probable photosystem II oxygen-evolving complex protein 2	58.3	62	21.1	8.92 × 10^-4^
115488344	Photosystem I reaction center subunit XI, chloroplast	30.8	13	19.1	3.57 × 10^-2^
115472753	Chlorophyll a/b-binding protein	49.0	39	18.7	8.07 × 10^-4^
115477831	Chloroplast photosystem I reaction center subunit II-like protein	59.1	50	18.4	3.46 × 10^-7^
115476576	Putative chlorophyll a/b-binding protein	36.5	27	14.5	2.05 × 10^-2^
115458738	OSJNBa0036B21.6 protein	38.5	19	13.7	2.74 × 10^-4^
115484899	Chlorophyll a/b-binding protein	63.2	67	13.3	1.17 × 10^-5^
115470199	PsbQ domain protein family, putative-like protein	28.4	11	10.9	1.53 × 10^-3^
115472785	Putative chlorophyll a/b-binding protein of LHCII type III, chloroplast	50.4	20	10.4	4.90 × 10^-3^
115446893	Putative Oxygen-evolving enhancer protein 3-2, chloroplast	26.2	6	10.1	2.74 × 10^-2^
115487694	Photosystem I reaction centre subunit N, chloroplast	28.2	8	10.0	2.12 × 10^-2^
115450991	Ribulose-phosphate 3-epimerase, chloroplastic	50.0	28	7.8	8.74 × 10^-4^
115467828	Chlorophyll a/b-binding protein	31.1	27	7.8	6.31 × 10^-3^
115452127	Fructose-1,6-bisphosphatase, chloroplastic	38.9	45	6.8	5.54 × 10^-6^
115482366	PsbP family protein	18.1	16	5.6	1.14 × 10^-3^
115465942	Ferredoxin--NADP reductase, leaf isozyme, chloroplastic	49.7	66	5.4	5.64 × 10^-3^
115447507	Putative ferredoxin-thioredoxin reductase	20.1	4	2.5	2.91 × 10^-2^
	Defense response				
115458852	Bet v I allergen family protein	29.9	5	0.3	1.89 × 10^-3^
115452513	Pathogenesis-related protein 1	49.4	7	0.1	7.92 × 10^-4^
115489022	Pathogenesis-related protein	29.8	5	0.04	9.16 × 10^-4^
115489014	Pathogenesis-related protein PR10	25.6	4	0.03	1.88 × 10^-2^
	Proteolysis				
115470052	ATP-dependent zinc metalloprotease FTSH 1, chloroplastic	42.6	50	9.9	3.31 × 10^-8^
115453893	Membrane-associated zinc metalloprotease family protein	17.6	7	7.7	8.99 × 10^-4^
115489316	Eukaryotic aspartyl protease family protein	25.1	10	6.8	1.04 × 10^-2^
115447609	ATP-dependent zinc metalloprotease FTSH 7, chloroplastic	4.3	4	6.5	3.77 × 10^-2^
115480844	Serine carboxypeptidase family protein	13.3	8	5.6	3.69 × 10^-2^
115435898	ATP-dependent Clp protease proteolytic subunit	18.5	4	3.5	2.12 × 10^-2^
115450022	Oligopeptidase A-like	24.2	20	3.2	1.35 × 10^-4^
115452585	Probable glutamyl endopeptidase, chloroplastic	15.0	18	2.9	6.00 × 10^-3^
115488046	Serine carboxypeptidase 1	11.2	4	2.7	4.20 × 10^-2^
115444859	Peptidase aspartic	24.1	10	0.5	4.99 × 10^-2^
115437452	Ubiquitin carboxyl-terminal hydrolase	16.2	7	0.4	3.60 × 10^-2^
115482252	Ubiquitin-conjugating enzyme E2-23 kDa	20.1	3	0.4	1.14 × 10^-2^
115483755	Ubiquitin-activating enzyme E1 2	22.6	25	0.4	3.41 × 10^-3^
115463349	Putative DNA-binding protein GBP16	26.0	15	0.4	1.26 × 10^-2^
115454751	Proteasome subunit beta type-2	30.2	9	0.4	2.65 × 10^-2^
115465685	Putative serine carboxypeptidase	24.9	12	0.4	4.19 × 10^-3^
115451123	Proteasome subunit alpha type-6	43.1	13	0.3	1.22 × 10^-3^
115456219	Leukotriene A-4 hydrolase homolog	20.8	12	0.3	6.69 × 10^-3^
115480143	Proteasome subunit beta type	36.3	7	0.3	1.63 × 10^-2^
115444057	Proteasome subunit alpha type-1	40.0	13	0.3	1.08 × 10^-2^
115440299	Putative insulin degrading enzyme	3.0	2	0.3	2.91 × 10^-2^
115440617	Proteasome subunit alpha type-3	39.4	10	0.3	4.04 × 10^-2^
115480019	Proteasome subunit beta type-1	28.1	6	0.2	7.50 × 10^-3^
115448935	Proteasome subunit beta type	40.7	12	0.2	6.34 × 10^-4^
115476300	Aminopeptidase M1-B	22.7	19	0.2	8.85 × 10^-5^
115461973	Aspartic proteinase	23.8	11	0.2	3.50 × 10^-3^
115445047	Aminopeptidase M1-A	18.8	17	0.2	1.04 × 10^-5^
115451209	Eukaryotic aspartyl protease family protein	11.4	4	0.2	2.37 × 10^-3^
	Protein transport				
115475569	Preprotein translocase subunit SECY, chloroplastic	4.1	3	5.5	1.76 × 10^-3^
115454153	SEC1 family transport protein SLY1	8.0	4	5.3	1.93 × 10^-2^
115451815	Translocase of chloroplast	20.2	7	3.1	1.42 × 10^-2^
115452177	Protein TOC75, chloroplastic	34.4	25	2.9	3.10 × 10^-6^
115435528	Importin-alpha re-exporter	5.5	2	0.5	7.40 × 10^-4^
115435714	GTP-binding protein	21.2	3	0.3	4.33 × 10^-2^
115463933	Putative GDP dissociation inhibitor	30.8	16	0.3	3.53 × 10^-5^
115454911	Coatomer subunit alpha-1	22.6	25	0.3	4.93 × 10^-5^
115461356	Clathrin light chain 1	14.6	3	0.2	4.25 × 10^-2^
115463119	Coatomer subunit delta-1	11.5	7	0.2	1.90 × 10^-3^
	Translation				
115480611	Cysteinyl-tRNA synthetase	11.8	6	8.7	1.93 × 10^-3^
115450395	50S ribosomal protein L11, chloroplast	38.1	13	7.2	2.71 × 10^-2^
115488938	Elongation factor Ts	25.6	45	6.7	1.06 × 10^-9^
115436768	Tyrosine--tRNA ligase	22.3	12	6.6	3.46 × 10^-3^
115472897	Ribosome-recycling factor, chloroplastic	31.2	17	6.6	1.13 × 10^-3^
115449027	Putative isoleucyl-tRNA synthetase	8.2	8	6.3	1.95 × 10^-6^
115470767	Probable polyribonucleotide nucleotidyltransferase 1, chloroplastic	9.6	10	6.1	4.16 × 10^-4^
115445399	Putative 50S ribosomal protein L21, chloroplast	25.7	7	5.8	1.12 × 10^-2^
115489150	60S ribosomal protein L2	30.6	11	4.8	6.71 × 10^-3^
115486501	Peptide chain release factor 1	17.8	7	4.7	4.88 × 10^-4^
50233964	30S ribosomal protein S2, chloroplastic	25.9	10	4.5	8.95 × 10^-4^
115438779	Peptide deformylase 1B, chloroplastic	16.4	5	4.1	4.50 × 10^-2^
115458788	OSJNBa0072F16.12 protein	21.3	5	3.9	4.05 × 10^-2^
115450427	50S ribosomal protein L5, chloroplastic	42.6	17	3.9	1.43 × 10^-3^
115448755	Putative histidine-tRNA ligase	6.6	4	3.8	4.60 × 10^-2^
115451609	50S ribosomal protein L15, chloroplast	29.7	11	3.5	3.81 × 10^-2^
115446545	Putative threonyl-tRNA synthetase	14.1	9	2.9	1.25 × 10^-6^
115439267	Met-tRNAi formyl transferase-like	20.7	6	2.8	2.49 × 10^-3^
115465593	Translation initiation factor IF-2	14.3	7	2.5	4.04 × 10^-2^
115463659	Putative chloroplast ribosomal protein L1	30.6	24	2.4	1.55 × 10^-3^
115487526	60S ribosomal protein L3	29.3	17	2.1	1.93 × 10^-2^
115447385	Lysine--tRNA ligase	14.5	9	1.5	4.36 × 10^-2^
115488928	Tryptophanyl-tRNA synthetase	21.8	7	0.5	4.03 × 10^-2^
115453877	40S ribosomal protein S3	44.7	14	0.5	2.32 × 10^-3^
115487104	40S ribosomal protein S16	27.5	6	0.4	1.16 × 10^-2^
115434960	Putative tRNA-glutamine synthetase	11.2	8	0.3	7.99 × 10^-3^
115473889	Elongation factor 1-beta	39.7	21	0.3	1.27 × 10^-2^
115486179	40S ribosomal protein S9	26.2	6	0.3	2.61 × 10^-3^
115475427	Putative 60S ribosomal protein L7	22.5	9	0.2	2.22 × 10^-2^
	Protein folding				
115444001	Putative uncharacterized protein P0576F08.31	16.7	6	22.9	1.64 × 10^-4^
115458444	GrpE protein homolog	26.6	9	18.2	1.12 × 10^-2^
115476198	Putative peptidyl-prolyl cis-trans isomerase, chloroplast	34.3	21	14.9	5.61 × 10^-5^
115449059	Putative 20 kDa chaperonin, chloroplast	46.3	9	8.2	1.49 × 10^-2^
115461585	Peptidyl-prolyl cis-trans isomerase	39.2	23	7.7	3.33 × 10^-3^
115460872	OSJNBb0079B02.1 protein	4.6	3	6.1	2.96 × 10^-2^
115467746	Trigger factor-like	39.5	27	4.8	1.77 × 10^-4^
115472829	Putative peptidyl-proly cis-trans isomerase protein	29.2	20	4.7	5.14 × 10^-5^
115448437	Putative protease IV	14.5	10	4.7	6.37 × 10^-3^
115472151	Peptidyl-prolyl cis-trans isomerase	23.3	5	4.6	4.48 × 10^-2^
115488160	60 kDa chaperonin alpha subunit	55.5	64	3.8	5.70 × 10^-5^
115473507	Receptor protein kinase	11.7	8	3.8	1.55 × 10^-2^
115466004	Putative chaperonin 60 beta	48.2	63	3.7	1.65 × 10^-3^
115475740	Putative uncharacterized protein OSJNBb0075O18.114	23.2	6	3.6	6.47 × 10^-3^
115465267	Serine/threonine-protein kinase SNT7	13.6	8	3.4	1.46 × 10^-2^
115448713	Peptidyl-prolyl cis-trans isomerase	34.3	11	3.1	8.95 × 10^-4^
115484731	ABC-1 domain containing protein	9.0	7	2.9	1.57 × 10^-2^
115441683	ABC1-like	5.3	3	2.8	4.11 × 10^-2^
115477014	Putative heat-shock protein	21.0	17	2.5	1.11 × 10^-2^
115463261	Putative DnaJ protein	25.3	14	2.5	4.82 × 10^-3^
115487998	70 kDa heat shock protein	45.4	60	2.3	1.13 × 10^-2^
115469982	Endoplasmin homolog precursor	26.7	28	0.5	1.62 × 10^-2^
115456045	T-complex protein 1, theta subunit	34.1	17	0.4	1.77 × 10^-2^
115462083	Chaperonin protein	19.4	11	0.3	3.37 × 10^-2^
115471369	Calreticulin	19.8	9	0.2	1.11 × 10^-2^
115477393	Putative 70 kDa peptidylprolyl isomerase	15.3	9	0.2	3.70 × 10^-4^
115468394	T-complex protein 1 subunit gamma	21.3	12	0.2	1.36 × 10^-3^
115458184	Calnexin	26.6	15	0.2	4.69 × 10^-4^
	Monosaccharide metabolism				
115458768	Glyceraldehyde-3-phosphate dehydrogenase	63.4	120	22.5	1.98 × 10^-4^
115484401	Fructose-bisphosphate aldolase, chloroplastic	74.0	126	22.1	4.10 × 10^-7^
115468886	Fructose-bisphosphate aldolase	57.3	49	20.5	8.04 × 10^-7^
115455637	Malate dehydrogenase	67.0	35	12.6	8.32 × 10^-4^
115450493	Glyceraldehyde-3-phosphate dehydrogenase	57.2	91	7.7	2.32 × 10^-5^
115466256	Putative enolase	46.0	32	7.3	1.48 × 10^-2^
115470849	Putative ribose-5-phosphate isomerase	52.5	32	5.6	1.19 × 10^-2^
115477891	PfkB type carbohydrate kinase protein family-like	12.1	4	5.3	1.34 × 10^-2^
115434516	Triosephosphate isomerase, cytosolic	69.2	29	5.0	4.75 × 10^-2^
115462281	Fructose-6-phosphate 2-kinase/fructose-2,6-bisphosphatase	22.9	20	5.0	8.62 × 10^-8^
115479643	Glucose-6-phosphate isomerase	29.9	19	4.8	3.56 × 10^-4^
115457638	OSJNBa0023J03.8 protein	31.3	8	4.5	2.00 × 10^-2^
115455133	4-hydroxy-3-methylbut-2-enyl diphosphate reductase, chloroplastic	35.9	19	3.5	7.98 × 10^-4^
115437808	Oxidoreductase-like	24.3	8	2.3	3.35 × 10^-2^
115464965	Hexokinase-5	24.1	10	2.2	2.59 × 10^-2^
115439869	Hexokinase-6	27.1	14	2.1	1.22 × 10^-2^
115452337	L-ascorbate peroxidase 1, cytosolic	49.2	29	0.4	9.02 × 10^-3^
115467370	Putative pyrophosphate-dependent phosphofructokinase beta subunit	31.2	17	0.4	9.90 × 10^-3^
115484175	Pyruvate kinase	31.3	23	0.3	2.35 × 10^-2^
115465974	6-phosphogluconate dehydrogenase, decarboxylating 1	44.8	29	0.3	6.70 × 10^-7^
115434198	Fructose-bisphosphate aldolase	24.5	10	0.3	1.53 × 10^-2^
115441963	Putative transaldolase	42.6	22	0.2	2.29 × 10^-3^
115473973	Xylose isomerase	34.5	17	0.2	1.06 × 10^-5^
	Disaccharide metabolism				
115439937	Putative trehalose-6-phosphate synthase/phosphatase	5.0	5	4.1	2.00 × 10^-2^
115452927	Sucrose synthase 4	9.4	9	0.5	1.24 × 10^-2^
115466896	Sucrose synthase 2	36.1	32	0.4	1.10 × 10^-3^
115453437	Sucrose synthase 1	43.3	36	0.1	2.55 × 10^-5^
	Polysaccharide metabolism				
115471703	Granule binding starch synthase II	22.2	14	25.6	7.88 × 10^-5^
115474235	Putative uncharacterized protein P0034A04.101-1	26.4	30	17.4	5.07 × 10^-5^
115451283	Inositol-3-phosphate synthase	23.7	11	9.2	4.04 × 10^-5^
115476014	Glucose-1-phosphate adenylyltransferase small subunit, chloroplastic/amyloplastic	36.1	23	6.4	7.94 × 10^-3^
115455167	Glucose-1-phosphate adenylyltransferase	42.7	30	4.4	4.71 × 10^-8^
115460666	Soluble starch synthase III-1	11.5	16	3.8	3.09 × 10^-2^
115461086	Probable UDP-arabinopyranose mutase 2	10.7	4	0.3	1.00 × 10^-2^
115470060	1,4-alpha-glucan-branching enzyme, chloroplastic/amyloplastic	7.3	6	0.1	1.93 × 10^-3^
115454033	UDP-arabinopyranose mutase 1	52.2	24	0.1	1.53 × 10^-6^
115459168	Chitinase 4	11.8	3	0.1	1.53 × 10^-2^
	Fatty acid metabolism				
115444801	Lipoxygenase	16.3	12	17.1	4.99 × 10^-6^
115489048	Lipoxygenase	17.6	15	7.0	7.04 × 10^-3^
115441871	Acyl-[acyl-carrier-protein] desaturase 2, chloroplastic	11.5	4	4.6	1.66 × 10^-2^
115436430	Putative tetrafunctional protein of glyoxysomal fatty acid beta-oxidation	17.3	13	0.3	2.76 × 10^-4^
115445513	Peroxisomal fatty acid beta-oxidation multifunctional protein	21.9	18	0.1	7.07 × 10^-8^
	Amino acid metabolism				
115455221	Serine hydroxymethyltransferase	57.1	73	22.1	4.47 × 10^-12^
115461066	Glutamine synthetase, chloroplastic	61.0	69	20.1	5.47 × 10^-4^
115460656	Aminomethyltransferase	57.1	51	19.8	4.34 × 10^-5^
115442595	Cysteine synthase	51.3	60	14.6	1.19 × 10^-4^
115439533	Glycine dehydrogenase P protein	60.8	157	12.8	1.08 × 10^-4^
115457070	Cysteine synthase	43.0	18	9.7	3.31 × 10^-5^
115478398	Aspartate kinase-homoserine dehydrogenase	10.9	11	5.8	2.85 × 10^-3^
115476972	Putative 3-deoxy-D-arabino-heptulosonate 7-phosphate synthase	23.6	12	5.2	2.76 × 10^-4^
115433966	Os01g0101200 protein	19.0	10	3.1	2.48 × 10^-2^
115480417	Putative dehydroquinate synthase	37.9	20	2.8	7.39 × 10^-3^
115450561	ATP phosphoribosyltransferase, chloroplastic	22.8	10	2.7	1.57 × 10^-2^
115448201	Carbamoyl-phosphate synthase small chain, chloroplastic	20.7	9	2.7	4.08 × 10^-2^
115445929	Probable diaminopimelate decarboxylase, chloroplastic	30.4	14	2.5	2.14 × 10^-3^
115486343	Phosphoserine phosphatase	17.6	4	2.5	4.98 × 10^-2^
115468570	Cysteine synthase	11.2	5	2.3	4.85 × 10^-2^
115482324	Glutamine synthetase family	4.9	4	0.6	3.26 × 10^-2^
115461214	Methylthioribose kinase 1	14.2	6	0.4	4.30 × 10^-2^
115449517	Glutathione reductase, cytosolic	20.8	9	0.4	2.44 × 10^-2^
115456165	Probable methylenetetrahydrofolate reductase	36.4	24	0.4	9.95 × 10^-6^
115466226	3-phosphoshikimate 1-carboxyvinyltransferase	22.7	12	0.4	3.98 × 10^-2^
115434790	Phospholipase D alpha 1	28.5	23	0.3	6.75 × 10^-4^
115454997	Glutamate decarboxylase	22.4	10	0.3	7.16 × 10^-3^
115447403	Phenylalanine ammonia-lyase	45.6	36	0.1	2.39 × 10^-2^
	Generation of precursor metabolites and energy				
115472339	Putative ATP synthase gamma chain 1, chloroplast	44.4	70	24.9	1.72 × 10^-9^
115472727	Cytochrome b6-f complex iron-sulfur subunit, chloroplastic	56.0	37	23.1	1.34 × 10^-4^
115457390	ATP synthase B chain	50.3	23	11.7	2.97 × 10^-3^
115435200	Putative phosphoenolpyruvate carboxylase 1	29.0	34	7.4	1.51 × 10^-4^
115452259	ATP synthase B chain, chloroplast	34.6	30	5.8	8.67 × 10^-4^
115448701	Putative H(+)-transporting ATP synthase	26.3	25	5.1	8.73 × 10^-4^
115469362	Putative vacuolar proton-ATPase	43.4	36	0.6	1.69 × 10^-2^
115435934	NAD-dependent isocitrate dehydrogenase a	29.3	11	0.6	3.96 × 10^-2^
115474559	Succinate dehydrogenase [ubiquinone] iron-sulfur subunit, mitochondrial	24.9	8	0.5	1.16 × 10^-2^
115438975	Putative H + -exporting ATPase	40.0	11	0.5	6.50 × 10^-3^
115444791	Citrate synthase	26.9	13	0.4	2.19 × 10^-3^
115447367	Succinyl-CoA ligase [ADP-forming] subunit beta, mitochondrial	31.0	14	0.3	1.77 × 10^-2^
115470583	Ferredoxin--NADP reductase, embryo isozyme, chloroplastic	16.4	6	0.3	5.45 × 10^-3^
115470493	Succinate dehydrogenase [ubiquinone] flavoprotein subunit, mitochondrial	13.2	9	0.2	2.91 × 10^-3^
115469332	Glutaredoxin-C8	36.4	3	0.1	4.59 × 10^-2^
115459340	Glutaredoxin-C6	43.8	7	0.1	9.69 × 10^-3^
115470941	Thioredoxin H1	40.2	11	0.1	7.29 × 10^-3^
	Vitamin metabolism				
115472485	Thiamine thiazole synthase, chloroplastic	49.8	29	6.7	5.04 × 10^-3^
115454593	Thiamine biosynthesis protein thiC	25.7	14	5.4	3.81 × 10^-7^
115446113	Riboflavin biosynthesis protein RibD family protein	9.2	4	3.9	1.81 × 10^-2^
115482032	GDP-mannose 3,5-epimerase 1	42.6	26	2.7	4.02 × 10^-2^
	Nucleotide metabolism				
115475007	Putative uncharacterized protein OJ1590_E05.35-1	10.5	4	9.5	7.33 × 10^-3^
115455473	WRKY DNA binding domain containing protein	4.9	5	5.1	1.59 × 10^-2^
115450117	(RAP Annotation release2) Formyltetrahydrofolate deformylase family protein	13.2	4	4.2	1.28 × 10^-2^
115462253	Probable GTP diphosphokinase CRSH2, chloroplastic	15.7	9	3.8	4.78 × 10^-2^
115480339	Deoxyribodipyrimidine photolyase family protein-like	8.5	6	3.5	1.70 × 10^-2^
115488968	Nucleoside diphosphate kinase	31.8	11	3.3	9.20 × 10^-3^
115454773	Adenylosuccinate synthetase 2, chloroplastic	34.0	21	3.1	8.43 × 10^-4^
115464251	Putative uracil phosphoribosyltransferase	28.9	9	3.0	7.42 × 10^-4^
115451155	SAP-like protein	13.1	4	2.9	4.14 × 10^-2^
	Isoprenoid metabolism				
115472641	Putative isopentenyl pyrophosphate:dimethyllallyl pyrophosphate isomerase	12.6	3	15.6	7.33 × 10^-3^
115447171	4-Hydroxy-3-methylbut-2-en-1-yl diphosphate synthase, chloroplastic	28.2	21	9.2	1.37 × 10^-8^
115471093	Zeta-carotene desaturase	26.8	18	7.9	4.21 × 10^-6^
115458652	Zeaxanthin epoxidase, chloroplastic	16.2	10	5.9	3.86 × 10^-5^
115434044	1-Deoxy-D-xylulose 5-phosphate reductoisomerase, chloroplastic	24.7	15	4.5	3.59 × 10^-2^
115451171	Phytoene dehydrogenase, chloroplastic/chromoplastic	15.4	9	2.8	2.84 × 10^-2^
	Phosphorus metabolism				
115463815	Pyruvate, phosphate dikinase 1, chloroplastic	40.4	51	7.4	1.85 × 10^-11^
115448919	Chloroplast inorganic pyrophosphatase	42.2	19	6.3	2.57 × 10^-2^
115488252	Phosphoglucan, water dikinase, chloroplastic	12.9	15	3.8	2.37 × 10^-6^
115468200	Alpha-glucan water dikinase	13.0	18	3.0	8.67 × 10^-5^
	Sulfur metabolism				
115456862	ATP sulfurylase	55.6	17	7.0	2.17 × 10^-3^
115472303	Probable 5′-adenylylsulfate reductase 1, chloroplastic	20.6	11	3.9	5.43 × 10^-4^
115450913	Glutathione reductase, chloroplast	31.0	20	3.3	1.25 × 10^-3^
	Macromolecule catabolic process				
115444937	26S proteasome regulatory particle triple-A ATPase subunit 6	30.9	16	0.4	3.81 × 10^-3^
115466690	Putative 26S proteasome regulatory particle triple-A ATPase subunit 5a	20.3	12	0.2	2.09 × 10^-3^
	Response to reactive oxygen species				
115446663	Probable L-ascorbate peroxidase 8, chloroplastic	27.2	31	6.7	2.50 × 10^-2^
115450521	Catalase	47.2	38	6.2	1.09 × 10^-2^
115477837	Superoxide dismutase [Cu-Zn], chloroplastic	54.0	28	5.4	1.30 × 10^-2^
115473833	Thioredoxin reductase NTRC	33.0	12	4.4	1.38 × 10^-5^
115477687	L-Ascorbate peroxidase	34.4	24	3.2	1.48 × 10^-2^
	Cofactor metabolism				
115479433	Formate-tetrahydrofolate ligase	29.4	25	3.0	4.12 × 10^-3^
115440827	ABC transporter subunit-like	13.2	8	2.7	1.62 × 10^-2^
115434288	Putative SufD	18.1	9	2.6	4.64 × 10^-2^
	Regulation of nitrogen utilization				
115477733	Putative NADPH-dependent reductase	41.2	18	7.4	2.14 × 10^-7^
115445203	Putative UOS1	30.3	19	6.9	1.72 × 10^-6^
115469824	Putative UOS1	23.3	13	5.5	6.31 × 10^-4^
115453029	Divinyl chlorophyllide a 8-vinyl-reductase, chloroplastic	24.2	11	4.6	6.54 × 10^-3^
	Cellular homeostasis				
115472057	Thioredoxin-like protein CDSP32, chloroplastic	29.9	13	10.1	2.89 × 10^-5^
115444771	Peroxiredoxin-2E-2, chloroplastic	63.1	34	7.7	6.91 × 10^-5^
115466906	Peroxiredoxin Q, chloroplastic	45.2	22	7.6	5.32 × 10^-4^
115446541	2-Cys peroxiredoxin BAS1, chloroplastic	56.3	36	5.2	3.44 × 10^-3^
115477793	Putative auxin-regulated protein	32.8	13	4.5	3.11 × 10^-2^
115436320	Dihydrolipoyl dehydrogenase	56.3	47	3.9	4.04 × 10^-5^
115435536	Peptide transporter protein-like	10.7	3	2.8	1.39 × 10^-2^
115471449	Putative uncharacterized protein OJ1370_E02.126	39.3	10	1.8	2.24 × 10^-2^
115464793	Thioredoxin	14.9	3	0.5	3.06 × 10^-2^
115479475	Protein disulfide isomerase-like 2-3	15.7	5	0.3	2.01 × 10^-2^
115462193	Protein disulfide isomerase-like 2-1	17.2	6	0.3	1.99 × 10^-3^
115455973	Thioredoxin H2-2	14.2	2	0.2	3.38 × 10^-2^
115484585	Protein disulfide isomerase-like 1-1	28.1	20	0.1	9.78 × 10^-8^
	Oxidation reduction				
115484891	Rieske [2Fe-2S] domain	35.0	18	13.7	3.63 × 10^-5^
115459670	NAD(P)H-quinone oxidoreductase subunit M, chloroplastic	39.1	14	11.5	6.11 × 10^-3^
115481490	Flavonoid 3′-hydroxylase	6.1	3	7.8	3.64 × 10^-2^
115476190	Putative oxidoreductase, zinc-binding	51.0	34	6.6	3.97 × 10^-6^
115476820	Nitrate reductase [NADH] 1	6.3	5	6.0	1.29 × 10^-2^
115477461	Moco containing protein	34.5	13	5.1	1.02 × 10^-3^
115482950	Aldo/keto reductase family protein	9.3	3	5.1	2.94 × 10^-3^
115454109	Oxidoreductase, aldo/keto reductase family protein	38.5	16	4.9	2.69 × 10^-4^
115476618	Glyceraldehyde-3-phosphate dehydrogenase	36.5	29	4.7	8.09 × 10^-3^
115443657	Putative ferredoxin-NADP(H) oxidoreductase	55.1	51	4.3	2.52 × 10^-3^
115484125	L-galactono-1,4-lactone dehydrogenase 1, mitochondrial	6.7	3	3.9	1.56 × 10^-3^
115446723	Glucose/ribitol dehydrogenase family protein	19.1	4	2.6	1.50 × 10^-2^
115477843	Putative malate dehydrogenase [NADP], chloroplast	21.5	13	2.5	1.35 × 10^-2^
115438082	Cytosolic aldehyde dehydrogenase	21.5	11	2.1	4.10 × 10^-2^
115487892	NADP-dependent oxidoreductase P2	17.9	6	1.8	2.31 × 10^-2^
115456131	Putative alcohol dehydrogenase	26.7	6	0.6	4.09 × 10^-2^
115443911	NADPH-dependent mannose 6-phosphate reductase	26.9	12	0.6	1.66 × 10^-2^
115482810	Malic enzyme	20.2	11	0.5	2.47 × 10^-3^
115460254	OSJNBa0009P12.34 protein	12.4	4	0.5	1.82 × 10^-2^
115478070	Putative NADPH-dependent retinol dehydrogenase/reductase	26.1	8	0.4	3.40 × 10^-2^
115484519	Aldehyde dehydrogenase	12.0	5	0.4	7.24 × 10^-3^
115479375	Aldehyde dehydrogenase	29.9	15	0.4	6.28 × 10^-3^
115463191	Superoxide dismutase [Mn], mitochondrial	32.9	13	0.3	3.01 × 10^-2^
115464645	Hypothetical protein	5.7	3	0.3	3.11 × 10^-2^
115434810	NADH-cytochrome b5 reductase	22.8	7	0.3	2.15 × 10^-2^
115451245	Oxidoreductase, zinc-binding dehydrogenase family protein	16.1	5	0.3	1.48 × 10^-2^
115478148	Isopenicillin N synthase family protein	5.2	2	0.2	7.89 × 10^-3^
115462115	Putative 1-aminocyclopropane-1-carboxylate oxidase	11.0	3	0.2	1.34 × 10^-2^
	Response to oxidative stress				
115445243	Class III peroxidase 29	38.9	20	39.8	3.19 × 10^-3^
115460338	Haem peroxidase family protein	32.9	20	4.5	4.36 × 10^-5^
115436084	Class III peroxidase 11	26.2	8	4.4	2.78 × 10^-2^
115474059	Peroxidase	47.0	19	0.3	1.30 × 10^-2^
115436300	Class III peroxidase 16	23.1	10	0.3	2.50 × 10^-2^
115456523	Salt tolerance protein	27.5	7	0.2	5.25 × 10^-4^
115459848	Glutathione peroxidase	33.9	10	0.2	4.64 × 10^-2^
115442403	Putative peroxidase	37.9	19	0.1	3.85 × 10^-4^
	Others				
115450080	Cell division inhibitor-like	20.9	14	5.4	2.53 × 10^-2^
115450329	Peroxisomal membrane protein 11-1	21.9	5	4.8	2.94 × 10^-2^
115452321	Ribosomal protein L10 containing protein	50.9	15	4.1	3.30 × 10^-4^
115439157	Two pore calcium channel protein 1	2.0	1	3.8	3.56 × 10^-2^
115457630	Phototropin-2	17.0	12	2.9	1.21 × 10^-4^
115474273	Phosphoinositide phospholipase C	27.3	15	0.5	4.60 × 10^-2^
115446411	RNA binding protein Rp120	29.6	29	0.5	1.61 × 10^-2^
115448225	GTPase activating protein-like	5.2	4	0.3	5.92 × 10^-3^
115453079	Villin-3	20.7	17	0.3	5.15 × 10^-3^
115451401	Mitochondrial outer membrane protein porin 5	49.1	21	0.3	4.79 × 10^-3^
115441759	Dolichyl-diphosphooligosaccharide--protein glycosyltransferase subunit 2	10.3	6	0.3	1.12 × 10^-4^
297601526	Probable linoleate 9S-lipoxygenase 4	22.4	15	0.2	2.78 × 10^-3^
115434036	Putative isoflavone reductase	19.8	5	0.2	1.38 × 10^-2^
115486998	Non-specific lipid-transfer protein 2B	57.3	17	0.1	4.26 × 10^-3^
115444635	Response regulator	2.1	3	0.02	2.08 × 10^-2^

Note: “Peptides (95 %)” indicates distinct peptides were identified with at least 95 % confidence (protein score cutoff > 1.5); “Cov (95)” means percentage of matching amino acids from identified peptides with confidence over 95 %; Ratio and *P*-value represents tag labeled for mock leaves: tag labeled for RSV-infected leaves. Ratio >1.5 is considered as downregulated and <0.67 is upregulated

#### Chloroplast group

**Fig. 5:**
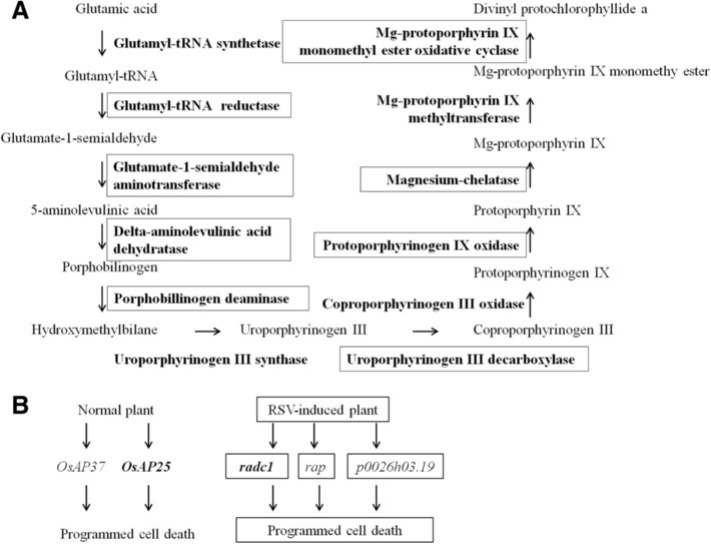
**a** Enzymes of chlorophyll biosynthetic pathway that decreased in accumulation during RSV infection. Selected steps are from KEGG pathways map (map 00860) for metabolism and enzymes. Bold words represent enzymes: glutamyl-tRNA synthetase, uroporphyrinogen III synthase, Mg-protoporphyrin IX methyltransferase, coproporphyrinogen III oxidase; boxed words represent enzymes: glutamyl-tRNA reductase, glutamate-1-semialdehyde aminotransferase, delta-aminolevulinic acid dehydratase, porphobillinogen deaminase, Mg-protoporphyrin IX monomethyl ester oxidative cyclase, magnesium-chelatase, protoporphyrinogen IX oxidase, uroporphyrinogen III decarboxylase. Eight enzymes at first stage of chlorophyll biosynthetic process were found and comprised 10 differentially accumulated proteins that were identified in RSV-induced leaves compared with the mock control leaves. **b** Two pathways could lead to programmed cell death including normal and RSV-induced plant. *OsAP25* (*radc1,* Os03g0186900), *OsAP37*, *rap*, and *p0026h03.19* were aspartic proteases genes

The 30 annotated significantly downregulated proteins in the chloroplast group process were involved in chlorophyll biosynthesis and photosynthesis (Table 2). For chlorophyll biosynthesis, 10 proteins involved in the chlorophyll contents in RSV-infected leaves were more than 3 times lower than in the mock leaves: magnesium chelatase subunit I (CHLI) and subunit D (CHLD), magnesium-protoporphyrin IX monomethyl ester [oxidative] cyclase, uroporphyrinogen decarboxylase 1, uroporphyrinogen decarboxylase 2, protoporphyrinogen oxidase, porphobilinogen deaminase, delta-aminolevulinic acid dehydratase, glutamate-1-semialdehyde 2,1-aminomutase, glutamyl-tRNA reductase (Table 2; Fig. 5). Twenty photosynthesis proteins were also annotated as enriched, whereas four oxygen-evolving enhancer proteins and a type protein involved in the chloroplast biosynthesis were over 10 times lower upon RSV infection than those in the mock control. Meanwhile, five chlorophyll a/b-binding proteins were downregulated in RSV-infected leaves compared with mock leaves (Table 2). Thus, the accumulation of 30 proteins in the chlorophyll metabolism was apparently reduced by RSV infection.

#### Defense group

Leaves are the primary tissue for RSV infection and colonization, so not surprisingly, four defensive proteins in RSV-infected leaves were identified as being altered in accumulation. Three pathogenesis-related proteins and a Bet v 1 allergen family protein were significantly more abundant in RSV-infected leaves than those in mock leaves: pathogenesis-related protein 1, pathogenesis-related protein 10, pathogenesis-related protein and Bet v I allergen family protein (Table 2). The upregulation of those proteins indicated that defensive reactions were induced after inoculation with RSV. From the 70 kDa heat shock protein (HSP70) family, ubiquitous in plants in response to diverse DNA and RNA viruses [39, 40], HSP70 and HSP (putative heat shock protein) were expressed at high levels in RSV-infected leaves compared with mock leaves, indicating that RSV activates the expression of the genes encoding HSP. In addition, superoxide dismutase [Mn] and four peroxidases expressed were upregulated in response to RSV (Table 2).

Of 28 annotated proteins involved in proteolysis, 19 proteins increased in response to RSV infection: 7 proteasome subunits, 3 ubiquitin type proteins, 3 aspartic type proteins, 2 aminopeptidase M1 subunits, 1 DNA-binding protein, 1 leukotriene A-4 hydrolase,1 serine carboxypeptidase and 1 insulin degrading enzyme. Three aspartic type proteins (eukaryotic aspartyl protease family protein, aspartic proteinase and peptidase aspartic) were expressed at a high level in the RSV-infected leaves (Table 2).

### Validation of changes in RNA level by RT-qPCR and Northern blotting

**Fig. 6:**
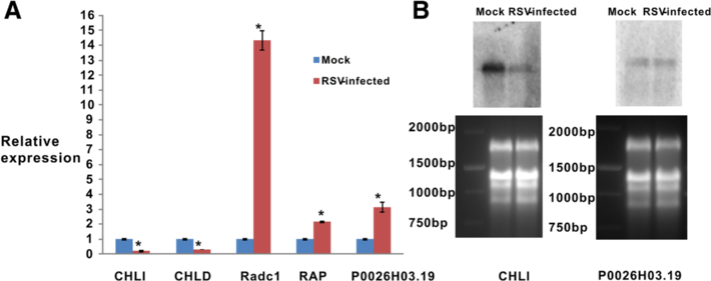
Validation of rice gene expression levels by real time RT-PCR and Northern blotting. **a** Comparison of protein and mRNA expression levels of mock leaves and RSV-infected leaves using RT-qPCR. Blue represents mock leaves; red represents RSV-infected leaves. The averaged readings from the three biological replicates normalized against endogenous gene *Os*EF1α; error bar denoted SD. Statistics were analyzed using the Student’s *t*-test. An asterisk indicated a significant difference from the corresponding control (*P* < 0.01). **b** Northern blot of two differentially expressed genes selected for verifying RT-qPCR results. Mock, mock-inoculated leaves; RSV-infected, RSV-infected leaves. Equal loading of total RNA was assessed by staining rRNA with ethidium bromide. Marker contained 2000 bp, 1500 bp, 1000 bp and 750 bp

Based on a proteomics analysis, the proteins differentially accumulated during RSV infection, key proteins for chlorophyll biosynthesis and an aspartic-type endopeptidase were identified as involved in the formation of RSV induced symptoms, and their presence was quantitatively confirmed using RT-qPCR and Northern blot to evaluate the correlation between mRNA and protein levels. Total RNA extracted from RSV-infected and mock leaves was analyzed to measure mRNA transcription levels of putative target proteins. The RT-qPCR results demonstrated that expression of the genes for CHLI and CHLD (magnesium chelatase) in RSV-infected leaves was downregulated more than three times the level of the control (Fig. 6a), and transcription of genes encoding radc1, rap and p0026h03.19 in RSV-infected leaves were upregulated 14, 2, 3 times higher than the level of the control leaves, respectively (Fig. 6a), verifying the iTRAQ results. Similarly, this trend for mRNA levels of the genes for CHLI and p0026h03.19 by Northern blotting analyses also supported the transcription of genes encoding respective protein by RT-qPCR (Fig. 6b). Whereas, elevated levels of five genes were different between transcription and proteins levels that may be due to posttranscription and posttranslational regulatory processes.

## Discussion

In the present study, iTRAQ-based experiments were implemented to identify proteins that were differentially accumulated between the RSV-infected and mock-inoculated leaves, then to determine which proteins may be involved in symptom formation. During RSV infection, 681 differentially accumulated proteins were found (Fig. 2; Table 1); 492 of these proteins were annotated by GO and located mostly in plastids, including the chloroplast, and participating in chlorophyll metabolism (Fig. 3, 4; Table 2). Chloroplast proteins was degraded by chloroplast vesiculation [41]. Upon RSV infection, the chloroplast vesiculation possibly targeted and destabilized the chloroplast for protein degradation, which resulted in cell death and induced the formation of vesicle containing many plastid proteins. According to the String database, protein-protein interaction networks were clustered in the chloroplast, defensive and metabolism groups (Additional file 2: Figure S1). Based on the functional analysis and RSV-induced disease symptoms, several proteins were associated with leaf chlorosis, cell death and plant defense during RSV invasion (Fig. 1, 3, 4). Additionally, the transcription of genes encoding selected proteins using RT-qPCR and Northern blot analyses matched with iTRAQ results (Fig. 6). We will discuss these various changes in proteins with regard to their significance to disease symptoms.

### RSV induced a decrease in chlorophyll

At 21 dpi, chlorotic stripes on newly emerged leaves are typical on rice plants infected by RSV (Fig. 1). Chlorosis is correlated with a reduction in chlorophyll during infection with a virus [11]. Recently, chlorophyll structure was also confirmed to be altered by accumulation of RSV SP, and PsbP (oxygen-evolving complex protein) was shown to participate in the interaction between rice and RSV [16]. Similarly, we used iTRAQ to determine that the accumulation of four oxygen-evolving enhancer proteins in RSV-induced leaves was lower than in the control plants (Table 2); thus, reduced accumulation of oxygen-evolving enhancer protein is involved in interrupting chlorophyll production.

Chlorophyll production is also influenced independently by chlorophyll anabolic and catabolic reactions [42]. Here, eight enzymes involved in early steps of chlorophyll biosynthesis were identified as being lower in RSV-infected leaves than in the mock-inoculated leaves (Fig. 5a; Table 2), again implicating RSV infection in significantly inhibiting chlorophyll biosynthesis. One of these eight, magnesium chelatase, comprising three subunits (CHLI, CHLD, CHLH), is an important synthetic enzyme for chlorophyll *a* and chlorophyll *b* [43]. Specifically, subunits CHLI and CHLD were downregulated in RSV-infected leaves (Table 2, Fig. 3a) and had decreased mRNA levels (Fig. 6) compared with the control. These subunits are AAA+ proteins (ATPases associated with various cellular activities) and form a motor unit, which provides a structure for the functioning of magnesium chelatase [44, 45]. The reduced accumulation of CHLI and CHLD thus indicates that the function of magnesium chelatase in chlorophyll biosynthesis is also limited. These results suggest that the reduction of chlorophyll is associated with downregulation of magnesium chelatase during infection with RSV. Previous studies of CMV have shown that the yellow mosaic symptoms are induced by a domain of satellite RNA [46, 47]. Recently, small interfering RNA (siRNA) derived from this domain of satellite RNA was shown to mediate RNA silencing of the chlorophyll biosynthetic gene CHLI (magnesium protoporphyrin cheltase subunit I) and that CHLI mRNA is downregulated in the infected tobacco [12, 13]. The yellowing domain of CMV satellite RNA induces RNA silencing of chlorophyll biosynthetic gene by small interfering RNA [12, 13]. Unlike CMV, RSV does not have satellite RNA; so how does RSV regulate and alter the chlorophyll biosynthetic pathway and induce chlorosis? In addition, a reduction of chlorophyll a/b-binding protein was shown to cause a downregulation of chlorophyll accumulation [14]. Here, the level of five chlorophyll a/b-binding proteins was reduced during RSV infection (Table 2). Therefore, RSV infection disrupts chlorophyll biosynthesis.

### Proteases coincided with cell death

The ubiquitin-26S proteasome system targets intercellular regulators that have a central role in battling pathogens [48–51] and in leaf senescence [52]. Several of the 26S proteasome units rose in accumulation in RSV-infected leaves compared with mock leaves (Table 2), suggesting it might promote host defense, then induce cell death in rice to restrict pathogen spread.

At the end stage of RSV infection, rice leaves developed chlorotic stripes, then the whole leaf died (Fig. 1b). Cell death requires a series of appropriate proteases. For example, over-expression of *OsAP25* (Os03g0186900) and *OsAP37* encoding aspartic proteases induces programmed cell death [18]. Similarly, in this study aspartic proteases encoded by *radc1* (Os03g0186900), *rap*, and *p0026h03.19* in RSV-infected leaves were sharply upregulated compared with the control leaves (Figs. 4 and 5b), indicating that the expression of the genes encoding aspartic protease was induced by RSV infection and participated in programmed cell death. However, we found that the aspartic protease pathway in RSV-infected leaves contained three proteins (radc1, rap, and p0026h03.19) that differed from the aspartic proteases (OsAP25 and OsAP37) in the normal plant*.* The aspartic protease pathway induced by a pathogen might thus be a new biological process.

### Defense reaction during RSV infection

Pathogenesis-related protein is associated with systemic acquired resistance of plant against diverse pathogens [53]. RSV infection induced a plant defense response, as noted by the upregulation of the expression of the genes encoding rice pathogenesis-related proteins. Bet v1 allergen, a member of the ubiquitous family of pathogenesis-related plant proteins, acts as a plant steroid carrier and has ribonuclease activity, suggesting it might play a key role in the plant defense response against pathogens [54–56]. In RSV-infected leaves, three pathogenesis-related proteins belonging to the Bet v1 allergen family of proteins (OSJNBb0048E02.12) accumulated at a higher level than in mock leaves (Table 2). So the upregulation of Bet v1 allergen family proteins might improve the transport of a steroid such as a brassinosteroid and enhance ribonuclease activities against virus infection. In addition, the heat-shock protein HSP70 was more abundant in the RSV-infected leaves than in mock leaves (Table 2); thus RSV can induce HSP70 accumulation, as can various other RNA and DNA viruses [39, 40]. The expression of the genes encoding superoxide dismutase [Mn], superoxide dismutase [Cu-Zn] and peroxidase was also altered in response to RSV invasion (Table 2). Superoxide dismutase and peroxidase in plant were also identified as upregulated in response to TMV infection [57]. However, superoxide dismutase [Cu-Zn] was identified as downregulated during *Sugarcane mosaic virus* infection, showing that the regulation of superoxide dismutase can differ depending on the virus [58]. RSV infection thus clearly activated the accumulation of rice defense-related proteins, similar to the defense-related proteins such as PR10, HSP70 and peroxidase induced in rice infected by *Rice yellow mottle virus* (RYMV) that were identified using the 2-D method [59].

## Conclusions

In summary, comparative proteomics analysis using iTRAQ LC-MS/MS technology identified 448 downregulated proteins and 233 upregulated proteins in many metabolic pathways during RSV infection. Several pathways potentially involved in RSV-induced symptom were found, including chlorophyll biosynthesis, proteolysis and defense response. Although our investigation provides knowledge of key proteins associated with the RSV-induced symptom, gene function analysis is needed to further understand the roles of these proteins in symptom formation. Therefore, our findings may provide new clues for elucidating the molecular mechanisms underlying RSV-induced symptom formation.

## Methods

### Insect population, plant materials and inoculation

A SBPH (small brown planthopper) population was maintained on susceptible rice (*Oryza sativa* var. *japonica*) cultivar (cv.) Wuyujing 3 in a climate chamber at 26 °C and a photoperiod of 14 h light and 10 h dark [60]. Third instar SBPH nymphs were allowed to feed on RSV-infected rice plants for a 3-day acquisition access period (AAP), then maintained in the climate chamber through the 10-day latent period. Ten viruliferous SBPH were then allowed to feed for a 2-day inoculation access period on three-leaved seedlings of *Oryza sativa* cv. Aichiasahi that had been grown in plastic pots containing a greenhouse soil mixture (40 % soil, 30 % vermiculite, 30 % straw powder). Subsequently, seedlings infested with non-viruliferous SBPH were used in the same way as a mock control. After the inoculation access period, seedlings were sprayed with insecticide and were transferred to insect-free greenhouse at 28 °C to observe symptom formation daily.

### Sampling and RT-PCR (reverse transcription-polymerase chain reaction)

Samples were collected from both RSV-infected leaves and mock leaves at 21 dpi and immediately immersed in liquid nitrogen. Total RNA was extracted using Trizol reagent (Invitrogen Trading, Shanghai, China). M-MLV reverse transcriptase (Promega, Madison, USA) was used to reverse-transcribe 2 μg of the total RNA with gene-specific primers (Additional file 1: Table S1). PCR was performed in a final volume of 50 μL at 95 °C for 5 min, 32 cycles of 95 °C for 30 s, 57 °C for 45 s, 72 °C for 50 s. Amplified products were fractionated in a 1 % agarose gel.

### Protein extraction, digestion and iTRAQ labeling

**Fig. 7:**
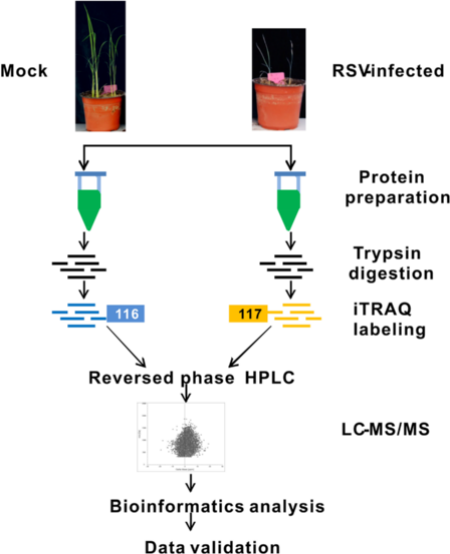
Strategy for iTRAQ LC-MS/MS analysis of comparative proteomics in rice infected with *Rice stripe virus* (RSV). At 21 days after inoculation, mock leaves (inoculated with healthy small brown planthoppers [SBPH]) and RSV-inoculated leaves (inoculated via RSV-viruliferous SBPH) were collected to extract total proteins. After prepared proteins were digested with trypsin, the peptides were labeled with the iTRAQ reagent and pooled. Pooled peptides were fractioned using the reversed-phase HPLC system, then individual fractions were analyzed using LC-MS/MS. MS raw data were processed using the NCBI protein database. Identified proteins were then analyzed using the DAVID platform and STRING software. Finally, key proteins were selected to validate their expression

To extract total proteins from the RSV-infected leaves and control leaves, the samples were homogenized in lysis buffer (7 M urea, 2 M thiourea, 0.1 % CHAPS), and the mixture was then incubated at 30 °C for 30 min, and centrifuged at 15,000 × *g* for 20 min at 4 °C. The supernatant was collected and the proteins concentration was determined by the Bradford protein assay (Bio-Rad Laboratory, Hercules, CA, USA). Bovine serum albumin (BSA) was performed as the standard for the calibration curve. Approximately 200 μg proteins were reduced with 1 M dithiothreitol, alkylated with 1 M iodoacetamide, dissolved in the dissolution buffer, and digested with trypsin (AB Sciex, Foster City, USA) at 1:50 (w/w) for 37 °C overnight, which were then labeled using the iTRAQ Reagents 4-plex kit (AB Sciex) according to the manufacturer’s instructions. The peptides from RSV-infected leaves and mock leaves were labeled with 117 and 116 tags, respectively (Fig. 7).

### Fractionation by reversed-phase high-performance liquid chromatography (HPLC)

Using the RIGOL L-3000 HPLC Pump system, the iTRAQ-labeled samples were reconstituted with mobile phase A (98 % H_2_O, 2 % acetonitrile, pH 10 adjusted by ammonia water) and mobile phase B (98 % acetonitrile, 2 % H_2_O adjusted by ammonia water), then fractionated on a Durashell-C18 column (4.6 mm × 250 mm, 5 μm, 100 Å; Agela, USA) at a speed of 0.7 mL min^-1^ using the gradient 0-5 min, 5-8 % buffer B; 5-35 min, 8-18 % buffer B; 35-62 min, 18-32 % buffer B; 62-64 min, 32-95 % buffer B; 64-68 min, 95 % buffer B; 68-72 min, 95-5 % buffer B. The chromatograms were recorded at 214 nm.

### Mass spectrometric (MS) analysis

The fractionated peptides, dissolved in 2 % methyl alcohol and 0.1 % formic acid were analyzed using an ABI-5600 system (Applied Biosystems). After equilibration of the column with solvent A (100 % H_2_O, 0.1 % formic acid), the peptides eluted from the column (EASY-Spray column, 12 cm × 75 μm, C18, 3 μm) with a 90-min mobile phase gradient using solvent B (100 % acetonitrile, 0.1 % formic acid) with a flow rate of 350 nL min^-1^, a spray voltage of 2.1 kV and ionization interface temperature of 250 °C. Scan range was from 350 to 1800 m/z. The charge states of peptides were set to +2 to +4.

### Protein identification and quantification

Proteins were identified and quantified by Protein Pilot Software 4.0 using the NCBI rice protein database (http://www.ncbi.nlm.nih.gov/protein/?term=oryza+sativa) search algorithm (Applied Biosystems). Proteins were identified based on various parameters such as trypsin digestion; dynamic modification; false discovery rate (FDR) determination for all peptide and protein identifications < 1 %; precursor ion mass tolerance, ±15 ppm; fragment ion mass tolerance, ±20 mmu; max missed cleavages, 2. Proteins were quantified as a change in relative expression; proteins with a fold-change >1.5 (*P* < 0.05) were considered to have decreased in level and those with fold-change <0.67 (*P* < 0.05) as increased.

### Bioinformatics analysis

The Gene Ontology (GO) annotation for functional analysis was done using the DAVID resources 6.7 (http://david.abcc.ncifcrf.gov/) [61], and proteins were classified based on the molecular function, biological process, and cellular components. The Kyoto Encyclopedia of Genes and Genomes (KEGG) (http://www.kegg.jp/) annotation was also done for a pathway analysis [62], and we assessed the interaction network for differentially accumulated proteins using STRING software (http://string-db.org/) [63].

### Analysis of gene expression by RT-qPCR (reverse transcription quantitative polymerase chain reaction)

RT-qPCR primers were designed by Primer Premier Version 5.0 based on the ORF (open reading frame) sequence of candidate genes cloned from rice (Additional file 1: Table S1), and a primer set for endogenous gene *Os*EF1α designed for another study [64] was also used. About 2 μg total RNA was reverse-transcribed using the FastQuant RT kit (Tiangen Biotech-Beijing Co.) according to the manufacturer’s instructions and then its concentration was measured by NanaDrop-1000 [65]. The RT-qPCR was done in final volume of 20 μL using the SupperReal PreMix Plus (SYBR Green) kit and the manufacturer’s instructions (Tiangen Biotech-Beijing Co.) in a ABI 7500 Real Time PCR thermal cycler and the following conditions: 95 °C for 15 min; 40 cycles of 95 °C for 10 s, 55 °C for 32 s, and 72 °C for 32 s. The experiment was repeated three times. Data for the melt curve were collected at 95 °C for 15 s, 60 °C for 1 min, 95 °C for 30 s, and 60 °C for 15 s. Relative gene expression was calculated by the 2^-ΔΔCT^ method [66].

### Northern blot analysis

Fifteen micrograms of the total RNA extracted was electrophoresed in a 1.5 % formaldehyde agarose gel and transferred to a Hybond-N^+^ membrane (GE Healthcare Bio-Scienes Corp., USA) [67]. The membrane was then baked at 80 °C for 2 h, then probed with α-^32^P-dCTP- randomly primer labeled probe at 65 °C overnight in a perfect hyb™ plus hybridization buffer (Sigma-Aldrich, St. Louis, USA). After the hybridization, the membrane was washed twice with 2× SSC (sodium chloride-sodium citrate), 1× SDS (sodium dodecyl sulfate); 1× SSC, 1× SDS and 0.5× SSC, 0.5× SDS at 65 °C, and the radioactive signals were detected using phosphor imaging.

## Additional files

Additional file 1: Table S1. The Gene Ontology (GO) annotation of differentially accumulated proteins using iTRAQ technology. Ratio represents tag labeled for mock leaves: tag labeled for RSV-infected leaves. Ratio >1.5 is considered as downregulated and <0.67 is upregulated. Additional file 2: Figure S1. The interaction network of differentially accumulated proteins between mock leaves and RSV-infected leaves using STRING soft program. We submitted 681 identified proteins to the STRING and analyzed 547 proteins in interaction with each other and constructing the network (A), which were roughly divided into three parts: metabolism (B), chloroplast (C) and defense (D).
