# Supplementary figures and images for: iTRAQ-based quantitative proteomics analysis of rice leaves infected by Rice stripe virus reveals several proteins involved in symptom formation
Source: Virol J. 2015 Jun 26;12:99. doi: 10.1186/s12985-015-0328-y (PMC4489111; doi:10.1186/s12985-015-0328-y)

A

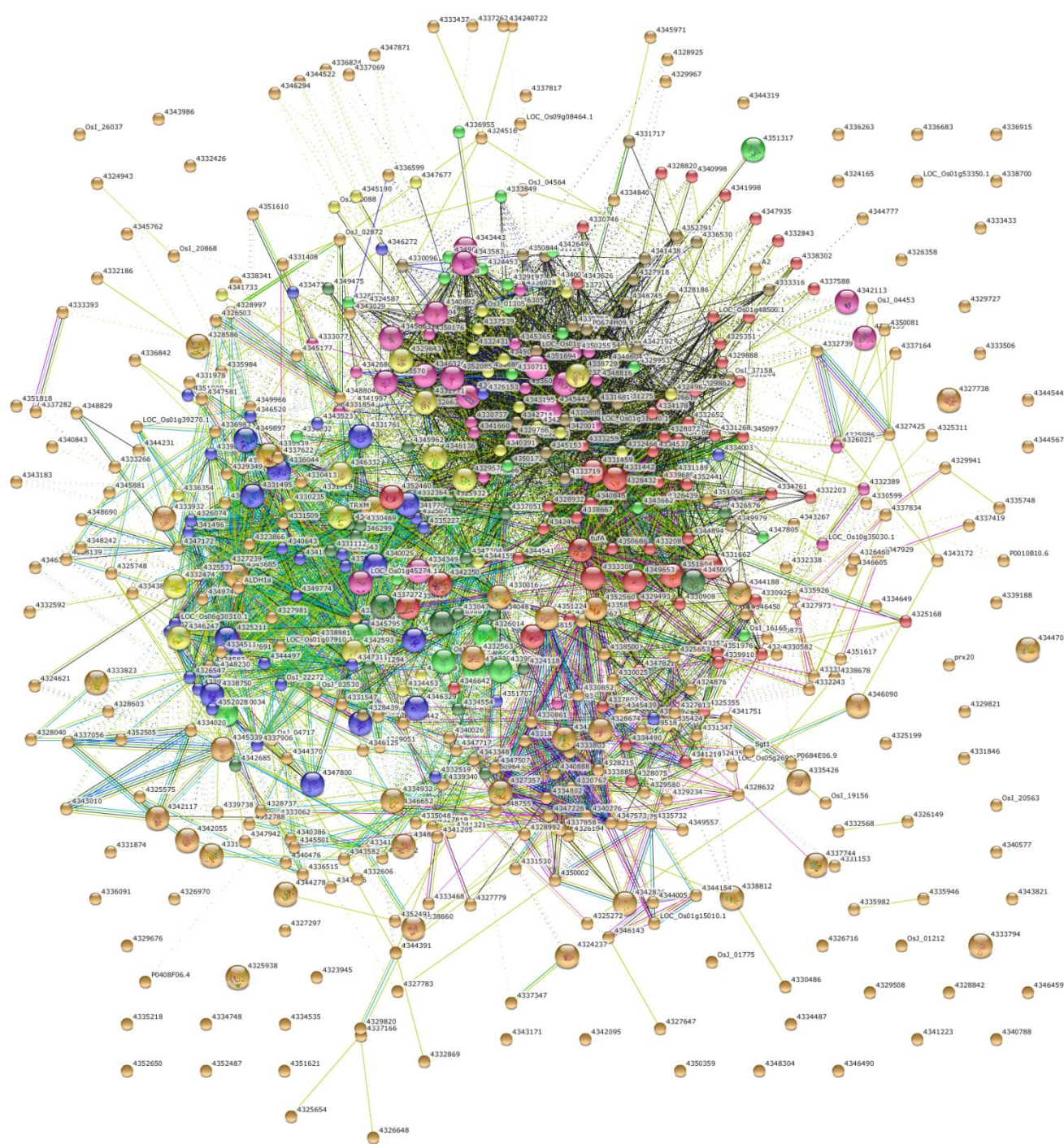

B

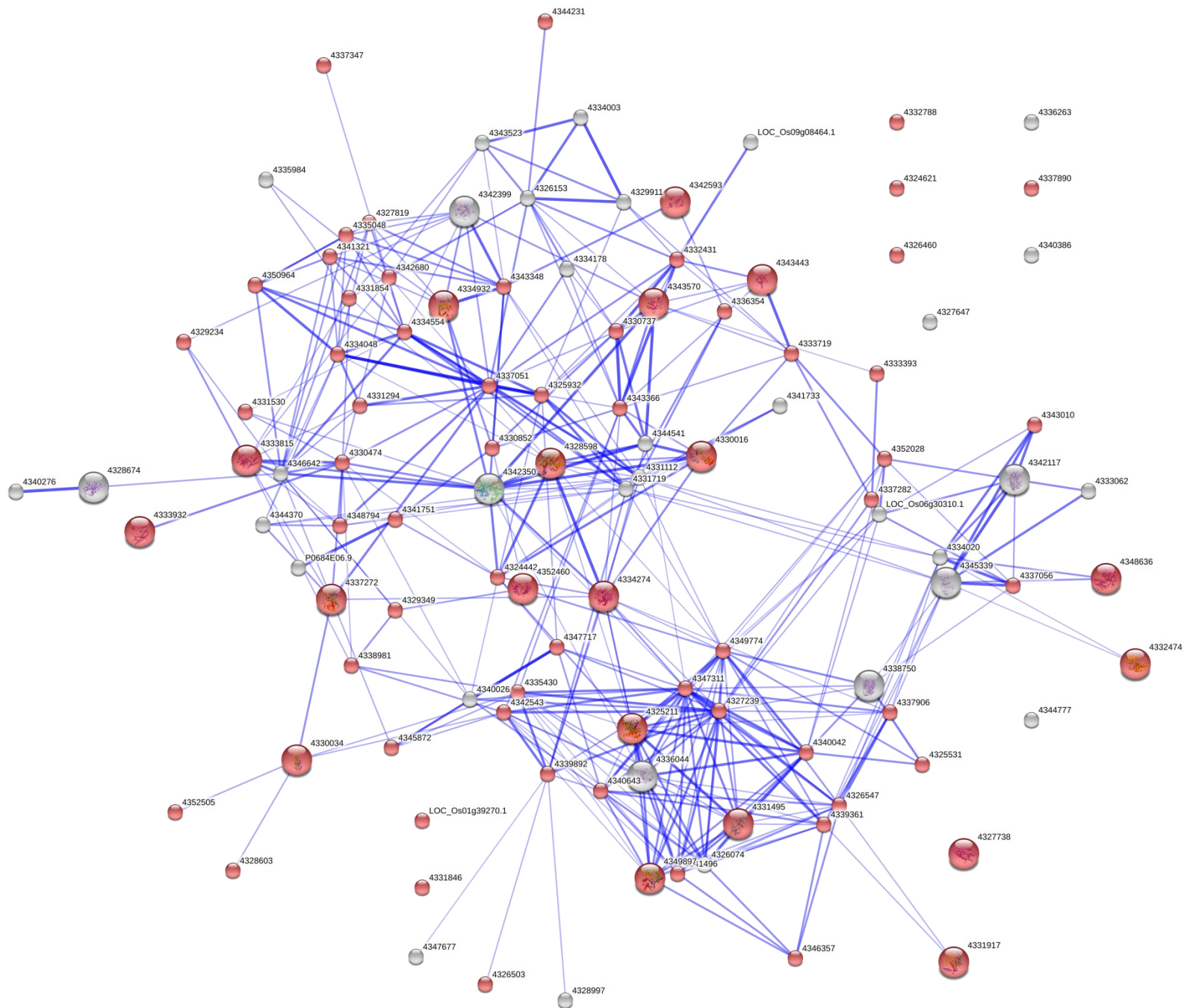

C

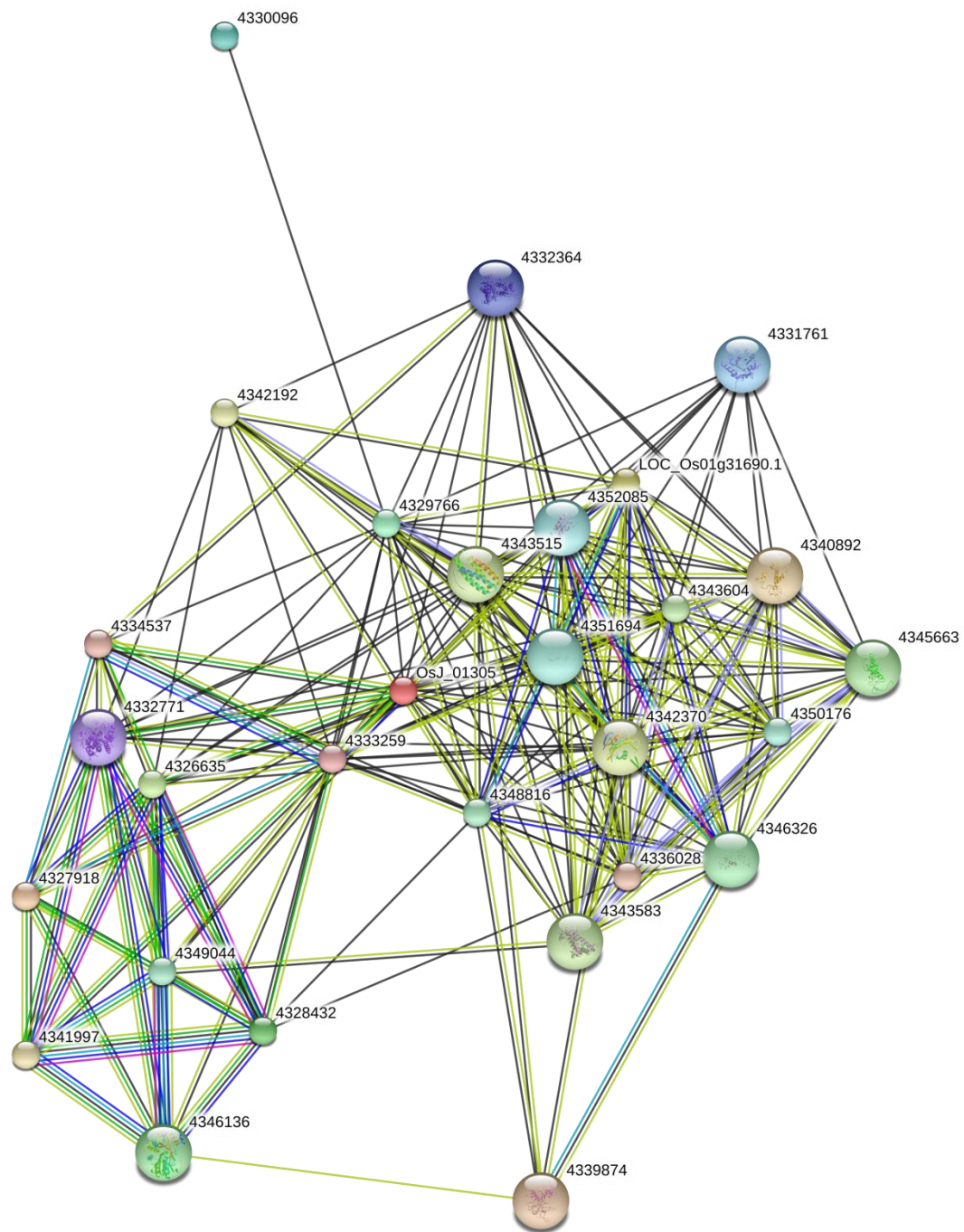

D

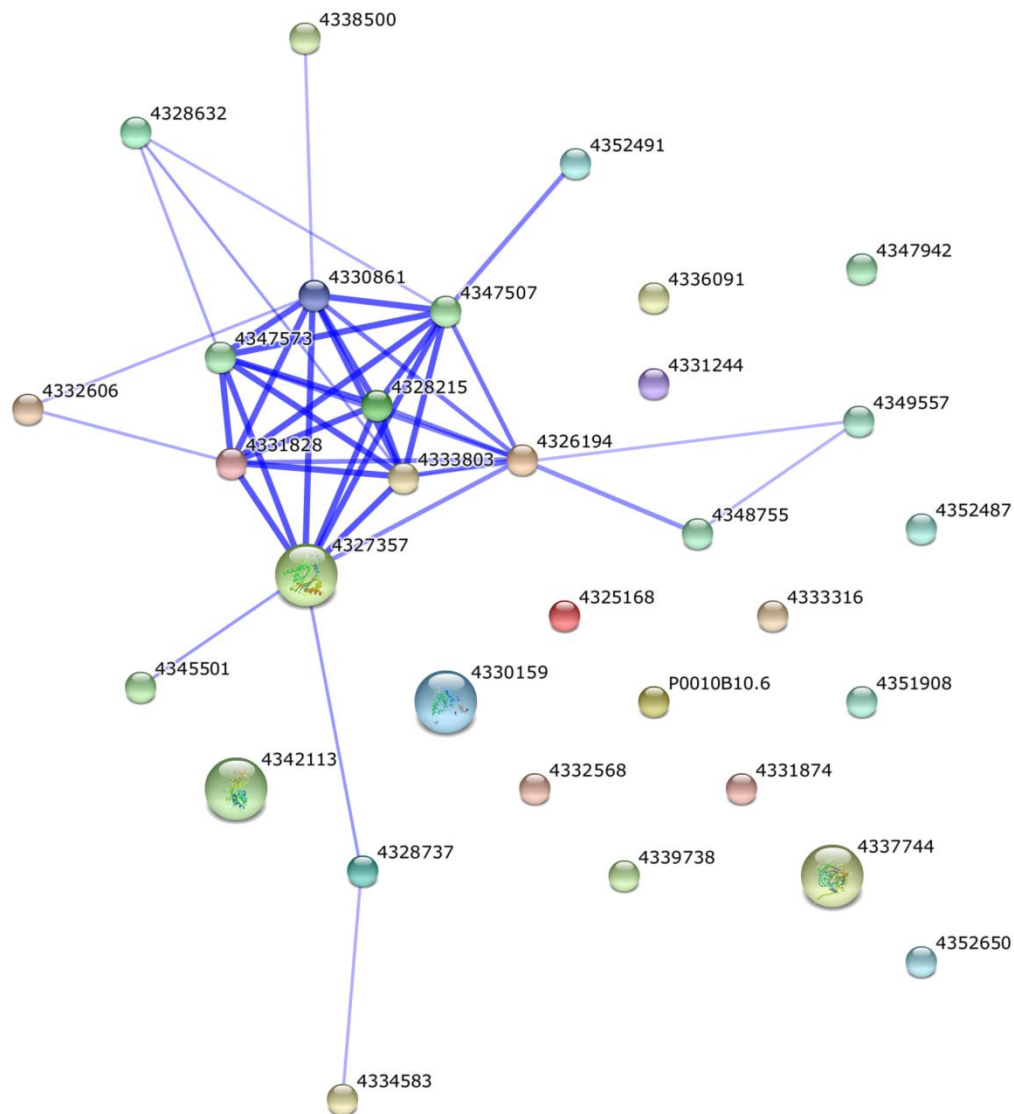

Supplement: Additional file 2: Figure S1. — The interaction network of differentially accumulated proteins between mock leaves and RSV-infected leaves using STRING soft program. We submitted 681 identified proteins to the STRING and analyzed 547 proteins in interaction with each other and constructing the network (A), which were roughly divided into three parts: metabolism (B), chloroplast (C) and defense (D). [file 12985_2015_328_MOESM2_ESM.pdf]
